# yApoptosis: yeast apoptosis database

**DOI:** 10.1093/database/bat068

**Published:** 2013-09-28

**Authors:** Kwanjeera Wanichthanarak, Marija Cvijovic, Andrea Molt, Dina Petranovic

**Affiliations:** ^1^Department of Chemical and Biological Engineering, Chalmers University of Technology, Kemivägen 10, 41296, Gothenburg, Sweden, ^2^Department of Mathematics, Chalmers University of Technology, Chalmers tvärgata 3, 41296, Gothenburg, Sweden, ^3^Department of Mathematics, University of Gothenburg, Chalmers tvärgata 3, 41296, Gothenburg, Sweden and ^4^Fine Chemicals and Biocatalysis Research, BASF SE, GVF/D - A030, 67056 Ludwigshafen, Germany

## Abstract

In the past few years, programmed cell death (PCD) has become a popular research area due to its fundamental aspects and its links to human diseases. Yeast has been used as a model for studying PCD, since the discovery of morphological markers of apoptotic cell death in yeast in 1997. Increasing knowledge in identification of components and molecular pathways created a need for organization of information. To meet the demands from the research community, we have developed a curated yeast apoptosis database, yApoptosis. The database structurally collects an extensively curated set of apoptosis, PCD and related genes, their genomic information, supporting literature and relevant external links. A web interface including necessary functions is provided to access and download the data. In addition, we included several networks where the apoptosis genes or proteins are involved, and present them graphically and interactively to facilitate rapid visualization. We also promote continuous inputs and curation by experts. yApoptosis is a highly specific resource for sharing information online, which supports researches and studies in the field of yeast apoptosis and cell death.

**Database URL:** http://www.ycelldeath.com/yapoptosis/

## Introduction

Yeast *Saccharomyces cerevisiae* (baker’s yeast or budding yeast) is a unicellular organism that has long been used as a common model organism for eukaryal cell and molecular biology. It has been extensively used to study many cellular processes such as cell metabolism, cell division and cell death. Apoptosis, one of the modes of programmed cell death (PCD), was initially extensively studied in mammalian cells, but it is also found in unicellular organisms, and was first described in yeast by Madeo *et al.* (1), where apoptotic markers were observed including DNA fragmentation, phosphatidylserine externalization and chromatin condensation. Other characteristic markers were described later in yeast including reactive oxygen species production (2) and cytochrome c release (3). Since this discovery, several studies have identified important genes and proteins in the yeast apoptotic pathways such as yeast orthologs of mammalian proteins (e.g. apoptosis-inducing factor, caspase and endonuclease G) (4), triggers and mechanisms that are involved in initiating or carrying out the apoptotic response (5). For more about yeast cell death, please check the following reviews (5–7).

More knowledge and data about yeast apoptosis are being discovered and generated, but the relevant information is dispersed in literature. Based on our own experience in this research field and feedback from the community, we identified the need for a repository platform to gather, organize and present the data and the research concepts. An example of a database in this area is the DeathBase (www.deathbase.org); however, its focus is on cell death proteins in metazoan cells (human, mouse, fly, worm and zebrafish) (8) and has limited use for yeast cell death research.

Here, we introduce an online database for yeast apoptosis, yApoptosis, that contains a list of apoptosis and PCD-related genes (called together apoptosis genes), curated apoptosis networks and protein complexes where these apoptosis genes participate and a network of co-regulated interacting proteins. This information can be accessed through the web interface with multiple functions.

## Aims of database

yApoptosis is a database dedicated to apoptosis genes, proteins and processes in yeast. The main aim of yApoptosis is to collect and organize apoptosis- and PCD-related information and to present it in a user-friendly format, so that it can facilitate collecting and searching for information about yeast cell death. We encourage participation from the research community to use yApoptosis as an online platform for communication, sharing and contributing useful information. Therefore, this platform will also provide opportunities for stronger collaborations in the field of yeast cell death research.

## Database implementation

yApoptosis is a database and a web interface. The data are stored and maintained using the database management system MySQL (http://www.mysql.com/). The web interface for browsing, searching and connecting to the back-end database was constructed on PHP (http://php.net/) and JavaScript (http://en.wikipedia.org/wiki/JavaScript). To provide interactive data visualizations including zoom, pan and clickable nodes, Google Maps API (https://developers.google.com/maps/) is used for graphical representation of the apoptosis networks (i.e. functional network, circuit network and clustered motif).

## Database content

**Figure 1.**
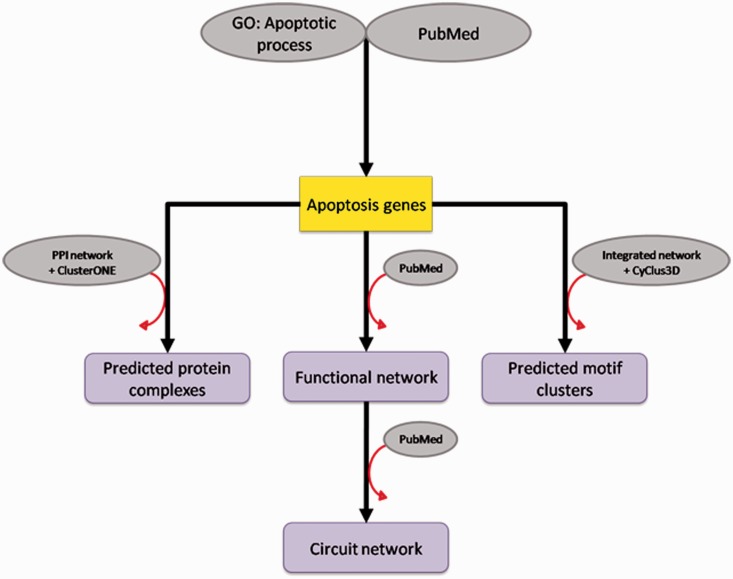
Data generation. From the defined GO term ‘apoptotic process' and searches of published data, the list of apoptosis-related genes was created. Some of those genes are illustrated in the functional network and its deducted version, the circuit network. The apoptosis-related genes were also used in the prediction of protein complexes and motif clusters where they participate.

In this section, we describe how we generated the database content that comprises a list of apoptosis-related genes and networks. The content is generated by an extensive curation and bioinformatics prediction (Figure 1).

### Apoptosis genes

**Figure 2.**
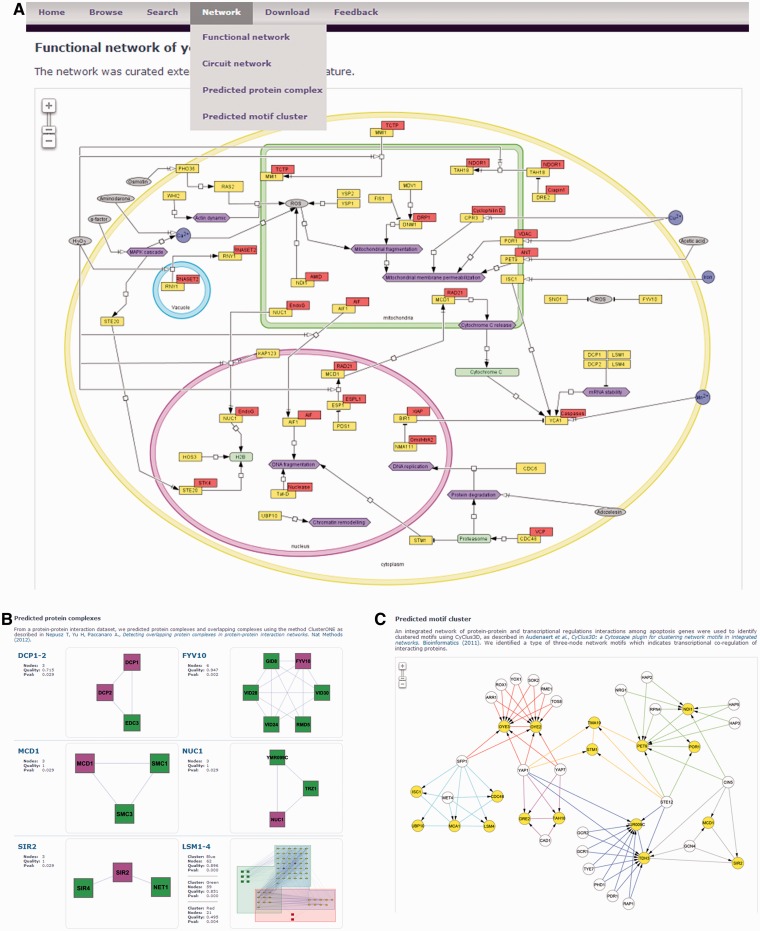
Networks of apoptosis genes. yApoptosis provides both static and interactive networks. (**A**) Snapshot of the functional network. (**B**) Snapshot of predicted protein complexes. (**C**) Snapshot of predicted motif clusters.

To be included into the database, a gene has to fulfill at least one of the following criteria: (i) its product (protein) is assigned with the gene ontology (GO) (9) term ‘apoptotic process’, (ii) it was found to directly regulate the basic machinery of apoptosis and (iii) it belongs to another pathway that induces apoptosis downstream. For each gene, general information such as gene description, pathway information, protein sequence, GO annotations, links to published literature supporting the role of this gene in apoptosis and crucial external links are provided. This information is manually curated. Brief details about each gene are based on information from the Saccharomyces Genome Database (SGD) (10) and are stored in the database. Pathway information indicates gene product localization and the process in which the gene product is involved in. Some of the genes were classified manually according to their localization and process they participate in as found in the literature search. Those genes are also illustrated in the functional network of apoptosis (Figure 2A). Besides, the pathway information is used as a filter of the ‘Search’ function. Additionally, for each gene yApoptosis provides extensive information via specific links to external resources: (i) Saccharomyces Genome Database for comprehensive integrated biological information, (ii) UniProt (11) for functional information on proteins, (iii) InterPro (12) for information on protein families and domains among genes that have a human homolog, (iv) PSICQUIC View (13) to retrieve molecular interaction data of a gene from multiple sources and (v) Gene Expression Atlas (14) to obtain expression profiles and conditions where a gene is differentially expressed.

### Networks of apoptosis genes

A network is a set of nodes connected by edges. For biological networks, nodes can represent different kinds of molecules (e.g. genes, proteins, metabolites, compounds, molecular complexes) or events (such as biological processes), whereas edges describe relationships between nodes, which include protein interactions, regulations, transport and conversions. In yApoptosis, to facilitate visualization of the apoptotic process and to show how apoptosis genes interact in different contexts, we graphically present four different networks (Figure 2).

### Functional network

The functional network of yeast apoptosis depicts activities and interactions/relations between apoptotic triggers (e.g. acetic acid), genes, proteins and processes in different locations or compartments (nucleus, mitochondria, cytoplasm and vacuole), which subsequently lead to apoptosis. The network was reconstructed based on extensive literature study and it was drawn using CellDesigner (15). Only genes that have pathway information are included in the network. The human orthologs of a particular gene are also represented (Figure 2A, red boxes). The network is available in different formats such as XML (http://en.wikipedia.org/wiki/XML) and SBML (16), which makes it suitable for directed uses in modeling software [e.g. Mathematica (http://www.wolfram.com/mathematica/), MATLAB (http://www.mathworks.se/products/matlab/) and COPASI (17)].

### Circuit network

The database also includes the circuit network [Kazemzadeh *et al.* (18)] that represents a deduced version of the functional network. The provided network can directly be used by simulation tools for Boolean analysis [SQUAD (19) and CellNetAnalyser (20)].

### Predicted protein complexes

To date, protein–protein interactions (PPIs) are the largest data sets available (21). The study of the protein interactome provides information on how these proteins assemble and work together. Densely connected regions in the PPI network represent protein complexes or modules that carry out particular roles (e.g. spliceosome). These complexes can be predicted computationally using several clustering algorithms [see (22) for review].

Hitherto, we have predicted the protein complexes in which a protein encoded by an apoptosis gene is a subunit of a complex. There are currently nine predicted complexes comprising products of 11 apoptosis genes (Figure 2B) and collected in the database. These complexes were predicted from the PPI network generated by Collins *et al.* (23) using the method ClusterONE (24). This data set combined two experimental yeast PPI data sets and calculated confidence of each PPI according to their purification enrichment score (23), and these interactions were used in our predictions. Assuming that proteins may have multiple functions, it is possible that proteins may belong to more than one complex. Therefore, we used the method ClusterONE, which not only can detect a protein complex but also can identify overlapping protein complexes. This method also takes into account edge scoring to improve the confidence of protein complex formation (24).

### Predicted motif clusters

In addition to PPI networks, there are various types of biological networks that include transcriptional regulations, genetic interactions, metabolic interactions and protein modifications (e.g. phosphorylation) (25). Combining such different networks into an integrated interaction network allows for studies of complex relationships between multiple interaction types.

Here, we first generated a yeast integrated network of protein–protein and transcriptional regulations interactions (26–28) and then retrieved all interactions for each apoptosis gene. The integrated network of yeast apoptosis contains 2698 interactions where 451 interactions are of the type transcriptional regulation and the rest are PPIs. CyClus3D (29) was used to identify network modules in the integrated network. The tool provides different network motifs for querying modules in the network. A network motif is a pattern of interactions that occurs frequently and significantly in complex networks. It describes a relationship between heterogeneous interaction types (29). Using this method, we identified a clustered motif that indicates transcriptional co-regulation of interacting proteins (Figure 2C), and most of them are in the same complex or act in concert.

### Community feedback and updates

Currently we provide a contact form and updates on the ‘Feedback’ page, which is a collaborative platform for users. In the future, if the research community expresses interest, we plan to propose an interactive forum or message board for discussions. So far, the updates have included corrections, curations and general comments about the improvement of yApoptosis, which are sent directly to the developer team.

## Data access

**Figure 3.**
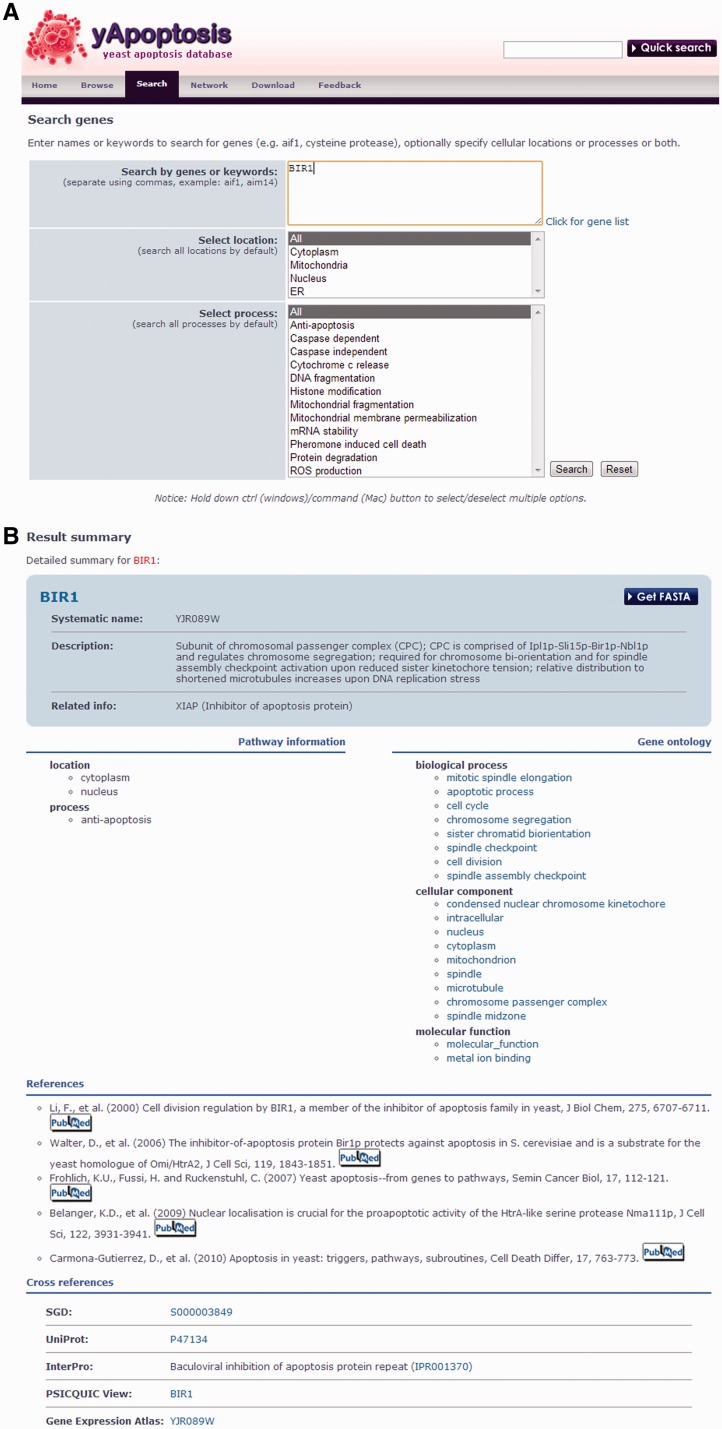
Screenshots of the yApoptosis web interface. (**A**) There are three main accessing methods: ‘browse’, ‘quick search’ and ‘search’. The search interface of yApoptosis shows that the database supports text-matching with the name or keyword of a gene and filtering with pathway information. (**B**) The ‘summary’ page contains general information as described in the text (example gene BIR1).

yApoptosis allows three main accessing methods to query for a particular apoptosis gene (Figure 3A): ‘browse’, ‘quick search’ and ‘search’. All genes stored in the database are listed on the ‘browse’ page, whereas ‘quick search’ and ‘search’ allow for a dynamic query with the gene name (i.e. systematic name, gene symbol or alias) or a keyword. The differences between two search functions are: (i) Search allows for searching by more than one gene or keyword, (ii) search allows the use of the empty text field returning all genes in the database and (iii) search includes alternative options to constrain the query by specifying pathway information, e.g. cellular location and process. Within the ‘browse’ and ‘search result’ table, the criteria for including the specific gene in the database are specified. Also, each gene has a dedicated ‘summary’ page (Figure 3B), which contains general information, as described earlier in text. In addition, genes can be accessed through the apoptosis networks on the ‘network’ page. All information in the database, including the networks, is available for download in different file formats on the ‘download’ page.

## Conclusions and future direction

yApoptosis is a user-friendly database dedicated to research of yeast cell death. To our knowledge, it is the first resource that structurally collects apoptotis and related genes of yeast and shares information through a user-friendly web interface. In addition to data from literature, we also include additional information based on computational predictions. Moreover, we provide a communication channel to encourage a collaborative online environment among researchers in the field.

The yApoptosis is the first part of yCellDeath (Yeast cell death database; http://www.ycelldeath.com), an online platform that will collect information on different aspects of yeast PCD. The plan is to continuously extend the database to include relevant data and information not only for apoptosis but also for other yeast cell death processes including necrosis, autophagy, stress and ageing. Moreover, relevant experimental data and recommended protocols will be integrated as additional information. To make it an even more powerful resource, it will incorporate bioinformatics tools for facilitating data analysis and visualization.

## References

[1] Madeo F, Frohlich E, Frohlich KU (1997). A yeast mutant showing diagnostic markers of early and late apoptosis. J. Cell Biol..

[2] Madeo F, Frohlich E, Ligr M (1999). Oxygen stress: a regulator of apoptosis in yeast. J. Cell Biol..

[3] Ludovico P, Rodrigues F, Almeida A (2002). Cytochrome c release and mitochondria involvement in programmed cell death induced by acetic acid in *Saccharomyces cerevisiae*. Mol. Biol. Cell.

[4] Mazzoni C, Falcone C (2008). Caspase-dependent apoptosis in yeast. Biochim. Biophys. Acta.

[5] Ludovico P, Madeo F, Silva M (2005). Yeast programmed cell death: an intricate puzzle. IUBMB Life.

[6] Munoz AJ, Wanichthanarak K, Meza E (2012). Systems biology of yeast cell death. FEMS Yeast Res..

[7] Carmona-Gutierrez D, Eisenberg T, Buttner S (2010). Apoptosis in yeast: triggers, pathways, subroutines. Cell Death Differ..

[8] Diez J, Walter D, Munoz-Pinedo C (2010). DeathBase: a database on structure, evolution and function of proteins involved in apoptosis and other forms of cell death. Cell Death Differ..

[9] Ashburner M, Ball CA, Blake JA (2000). Gene ontology: tool for the unification of biology. The Gene Ontology Consortium. Nat. Genet..

[10] Cherry JM, Hong EL, Amundsen C (2012). Saccharomyces genome database: the genomics resource of budding yeast. Nucleic Acids Res..

[11] Consortium U (2012). Reorganizing the protein space at the Universal Protein Resource (UniProt). Nucleic Acids Res..

[12] Hunter S, Jones P, Mitchell A (2012). InterPro in 2011: new developments in the family and domain prediction database. Nucleic Acids Res..

[13] Aranda B, Blankenburg H, Kerrien S (2011). PSICQUIC and PSISCORE: accessing and scoring molecular interactions. Nat. Methods.

[14] Kapushesky M, Adamusiak T, Burdett T (2012). Gene Expression Atlas update–a value-added database of microarray and sequencing-based functional genomics experiments. Nucleic Acids Res..

[15] Funahashi A, Tanimura N, Morohashi M (2003). CellDesigner: a process diagram editor for gene-regulatory and biochemical networks. BIOSILICO.

[16] Hucka M, Finney A, Sauro HM (2003). The systems biology markup language (SBML): a medium for representation and exchange of biochemical network models. Bioinformatics.

[17] Hoops S, Sahle S, Gauges R (2006). COPASI–a COmplex PAthway SImulator. Bioinformatics.

[18] Kazemzadeh L, Cvijovic M, Petranovic D (2012). Boolean model of yeast apoptosis as a tool to study yeast and human apoptotic regulations. Front. Physiol..

[19] Di Cara A, Garg A, De Micheli G (2007). Dynamic simulation of regulatory networks using SQUAD. BMC Bioinformatics.

[20] Klamt S, Saez-Rodriguez J, Gilles ED (2007). Structural and functional analysis of cellular networks with CellNetAnalyzer. BMC Syst. Biol..

[21] Koh GC, Porras P, Aranda B (2012). Analyzing protein-protein interaction networks. J. Proteome Res..

[22] Li X, Wu M, Kwoh CK (2010). Computational approaches for detecting protein complexes from protein interaction networks: a survey. BMC Genomics.

[23] Collins SR, Kemmeren P, Zhao XC (2007). Toward a comprehensive atlas of the physical interactome of *Saccharomyces cerevisiae*. Mol. Cell Proteomics.

[24] Nepusz T, Yu H, Paccanaro A (2012). Detecting overlapping protein complexes in protein-protein interaction networks. Nat. Methods.

[25] Zhu X, Gerstein M, Snyder M (2007). Getting connected: analysis and principles of biological networks. Genes Dev..

[26] Szklarczyk D, Franceschini A, Kuhn M (2011). The STRING database in 2011: functional interaction networks of proteins, globally integrated and scored. Nucleic Acids Res..

[27] Abdulrehman D, Monteiro PT, Teixeira MC (2011). YEASTRACT: providing a programmatic access to curated transcriptional regulatory associations in *Saccharomyces cerevisiae* through a web services interface. Nucleic Acids Res..

[28] Harbison CT, Gordon DB, Lee TI (2004). Transcriptional regulatory code of a eukaryotic genome. Nature.

[29] Audenaert P, Van Parys T, Brondel F (2011). CyClus3D: a Cytoscape plugin for clustering network motifs in integrated networks. Bioinformatics.

